# A Minimum Rank Approach for Reduction of Environmental Noise in Near-Field Array Antenna Diagnosis

**DOI:** 10.3390/jimaging5050051

**Published:** 2019-05-02

**Authors:** Marco Donald Migliore, Fulvio Schettino, Daniele Pinchera, Mario Lucido, Gaetano Panariello

**Affiliations:** Department of Electrical and Information Engineering, University of Cassino and Southern Lazio, Via Di Bisaio 43, 03043 Cassino, Italy

**Keywords:** antenna array, near-field measurements, 5G communication, array diagnosis, rank minimization, compressed sensing, antenna testing

## Abstract

A method to filter out the contribution of interference sources in array diagnosis is proposed. The interference-affected near field measured on a surface is treated as a (complex-data) image. This allows to use some modern image processing algorithms. In particular, two strategies widely used in image processing are applied. The first one is the reduction of the amount of information by acquiring only the innovation part of an image, as currently happens in video processing. More specifically, a differential measurement technique is used to formulate the estimation of the array excitations as a sparse recovery problem. The second technique has been recently proposed in video denoising, where the image is split into a low-rank and high-rank part. In particular, in this paper the interference field is filtered out using sparsity as discriminant adopting a mixed minimum $\ell_{1}$ norm and trace norm minimization algorithm. The methodology can be applied to both near and far field measurement ranges. It could be an alternative to the systematic use of anechoic chambers for antenna array testing.

## 1. Introduction

Sophisticated radiating systems as active array antennas, and massive MIMO arrays will play a relevant role in the forthcoming 5G communication systems [1]. Due to the high levels of electronic devices integration required in 5G antennas, no physical connectors are generally available. This yields a radically new connectorless measurement paradigm in which over-the-air (OTA) measurements will have a relevant role. Furthermore, mass production of these new antennas requires new, fast and reliable antenna testing methods. In this framework, near-field measurements represent the most interesting solution due to the accuracy and small dimension of the test set compared to far-field and compact range antenna measurement systems.

In near-field measurements [2] the field radiated by the Antenna Under Test (AUT) is measured on a scanning surface placed at short distance (5λ−7λ, λ being the free space wavelength) from the AUT in an anechoic chamber in order to avoid reflections and stray signals [3]. The propagation process from near-field to far-field conditions is simulated by a proper software. Even if the scanning area could be any sufficiently smooth surface, planar surfaces are most commonly adopted for array antennas.

The main advantages of near-field set-ups are well known: they allow to perform accurate measurements in a controlled environment. However, a further advantage, that is often underestimated, is the possibility of using sophisticated data processing algorithms to reduce the cost of the measurement process without affecting the accuracy. This possibility will be exploited in this paper with reference to array diagnosis.

On the other hand, near-field measurements suffer from two main drawbacks. The first one is the time required to collect the data on the observation surface using standard near-field set-ups [4]. With reference to this point, sparse recovery techniques have been recently proposed in the framework of antenna diagnosis from planar near-field measurements [5,6,7,8] in order to reduce the number of measured data and consequently the measurement time. The method has been successfully tested from data acquired in anechoic chamber [9,10].

A further problem is the cost of large anechoic chambers. Regarding this point, a large effort has been devoted to the reduction of the so-called truncation error, caused by a limited scanning area, in order to use smaller and less expensive anechoic chambers [11,12,13,14]. However, a further and more drastic solution is to perform measurements in a non anechoic environment, avoiding the use of expensive anechoic chambers.

The aim of this contribution is to investigate a technique that avoids the use of expensive anechoic chambers by filtering out the interference of undesired electromagnetic sources.

It is worth noting that other techniques for filtering interference signals in antenna measurements have been proposed. A partial list is reported in the references section [15,16,17,18,19,20]. The strategies followed in literature are based on a complete characterization of the environment in order to subtract the environment response [15], on the equivalent source reconstruction using inverse linear approaches [16], on the use of suitable base representations [19,20]. Large effort has also been devoted to interference filtering from amplitude-only measurements [17,18]. Generally speaking, these methods are ‘general purpose’, in the sense that they can be used in general near-field measurement systems, and do not take explicitly into account the small number of failures in array testing. In the proposed method this characteristic is explicitly exploited to reduce the set of possible array excitations, allowing to identify the failures and to filter the interference contributions in the same step.

The basic idea is to use differential measurements in order to obtain a sparse representation of the AUT excitations [21]. Such sparseness property of the radiating source is used as a-priori information in order to distinguish the AUT contribution from the contribution of scattering objects in measured data, that are characterized by a low rank field distribution.

It is worth noting that the method proposed in this paper strictly resembles the methods used in image processing. In practice, the interference-affected near field measured on a surface is treated as a (complex-data) image. This allows to use some modern image processing algorithms. In particular, two strategies widely used in image processing are applied in this paper. The first one is the reduction of the amount of information by acquiring only the innovation part of an image, as currently happens in video processing, using a differential measurements. The second technique has been recently proposed in video denoising, where the image is split into a low-rank and high-rank part [22]. In particular, in this paper the interference field is filtered out using sparsity as discriminant adopting a mixed minimum $\ell_{1}$ norm and trace norm minimization algorithm.

## 2. Rank and Sparsity of the Feld Radiated by an Electric Dipole

Before introducing the filtering technique, it is useful to briefly discuss the general idea at the base of the filtering procedure.

**Figure 1:**
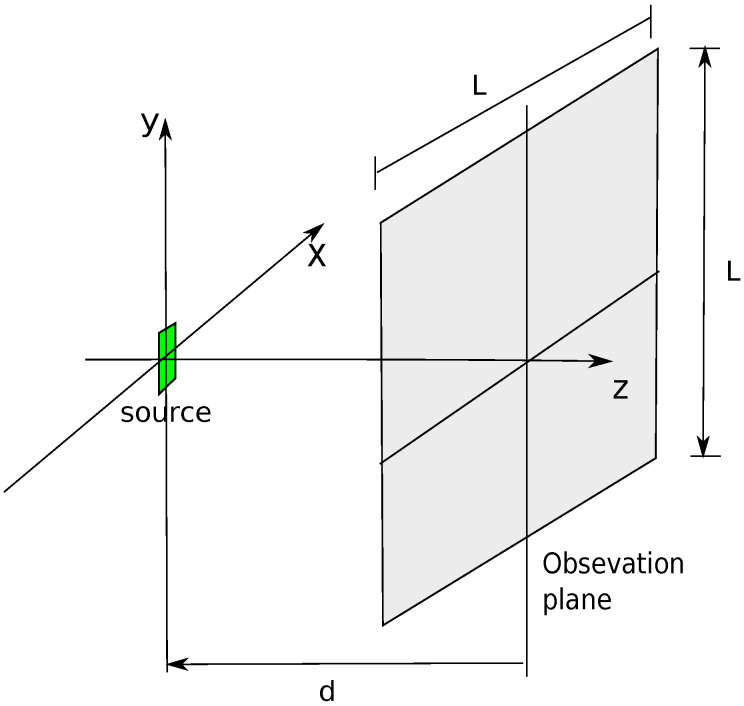
Geometry of the problem.

Let us consider a harmonic electromagnetic source consisting in an elementary electric dipole directed along the *y* direction (Figure 1). The dipole can model an element of an array, or also a scattering point caused by objects in the environment where the measurement system is placed. The field of this dipole is observed on a square surface having dimension L×L (L=20λ) placed on the z=d plane with a uniform planar grid at 0.2 λ sampling step. The field on the observation points is sampled and the data are collected on an equispaced grid and the measured values are collected in the matrix $\bar{X}$.

**Figure 2:**
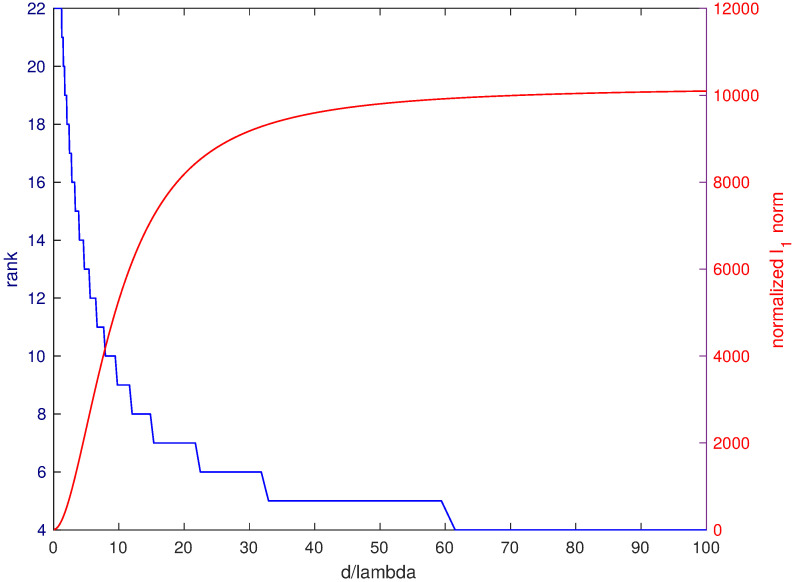
Blue curve (left scale): rank of the field on the observation plane; red curve (right scale): $\ell_{1}$ norm normalized to the maximum of the field amplitude on the observation plane; the observation plane is 20λ×20λ; *d* is the distance between the source point and the observation plane.

In Figure 2, the rank of the matrix $\bar{X}$ is evaluated versus the distance *d* between the source and the observation plane (blue curve, left scale). We can note that the rank rapidly decreases. As a consequence, at sufficiently large distance $\bar{X}$ tends to be a low rank matrix.

This ‘smoothing’ process in the propagation process is a general property of the field radiated by an electromagnetic source [23]. Loosely speaking, the propagation acts as a spatial low pass filter, smoothing the fast spatial variations of the field. Consequently, the field on the observation surface, being smoother, tends to require less basis functions for its representation.

The spreading of the field on the observation surface caused by the filtering property of the propagation phenomenon can also be quantified in terms of ‘sparseness’ of the field. For this purpose we should evaluate the so called $\ell_{0}$ norm, i.e., the number of elements of the matrix different from zero. The $\ell_{0}$ norm suffers from a number of drawbacks that prevent its use in practical problems. Instead of the $\ell_{0}$ norm, we will estimate the degree of sparsity using the 1-norm, or $\ell_{1}$ norm, of the matrix $\bar{X}$, i.e., the sum of all the amplitudes of the entries of the matrix. The more the field is concentrated on the observation surface, the smaller the 1-norm is. This point will be discussed in more detail in the next section. In this section the goal is to give an intuitive explanation of the usefulness of the $\ell_{1}$ norm.

The $\ell_{1}$ norm of the matrix, normalized to the maximum amplitude of the entries of the matrix, is plotted in Figure 2 versus the distance *d* between the source and the measurement plane (red curve, right scale). We can see that the normalized $\ell_{1}$ norm of the matrix increases rapidly with distance, as we should expect.

This simple example shows that rank and degree of sparsity can be used to distinguish contributions from sources in different positions provided that the plane is positioned in the right position. In particular, if the plane is placed very close to the ‘desired’ source, and sufficiently far from the interference source, it is possible to filter the undesired sources by subtracting the low rank contribution.

This observation is at the basis of the method proposed in this contribution to filter undesired field reflections.

## 3. The Array Failure Detection Algorithm with Reflection Filtering

**Figure 3:**
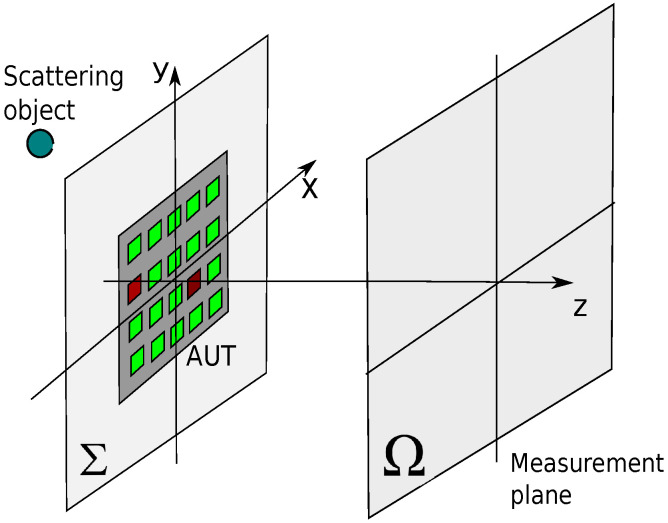
Measurement set-up; the data are collected on the surface Ω placed at a distance *d* from the AUT and are affected by a scattered field and Gaussian noise; some elements of the AUT are malfunctioning (red squares).

Let us consider an Antenna Under Test (AUT) consisting of a planar N×N array affected by a number of fault elements (Figure 3). Let Σ be the plane where the AUT aperture lies, and $X^{AUT}\in C^{N\times N}$ the matrix collecting the currents of the radiating elements of the AUT.

In differential measurements [5], we consider also an array without failures, called ‘golden array’, whose currents matrix is $X^{GOLD}$. $X^{GOLD}$ (as well as the field radiated by $X^{GOLD}$) can be obtained by full-wave numerical simulations, or by measurements in a controlled environment (i.e., in an anechoic chamber).

The field of the AUT and of the Golden array are measured on a plane Ω placed at distance *d* from the AUT in a square lattice of M×M points. The data are collected in the matrices $Y^{AUT}$ and $Y^{GOLD}$, and the following quantities

(1) $$\begin{array}{c} X=X^{AUT}-X^{GOLD} \end{array}$$

(2) $$\begin{array}{c} Y=Y^{AUT}-Y^{GOLD} \end{array}$$

are evaluated. Since the number of fault elements is much smaller than the number of elements of the array, the X matrix is *sparse* [24].

Now, let us suppose that there is an interference source and let us call $X^{s}$ the matrix collecting the equivalent currents generated by the field of the interference source on the plane Σ, i.e., the plane where the AUT is placed.

Consequently, the equivalent currents on Σ are given by the superposition of the equivalent currents associated to the AUT and to the interference source, i.e., $X^{AUT}+X^{s}$. Note that on this plane $X^{s}$ tends to be a low rank matrix as discussed in the previous section.

The field measured on the observation surface is the superposition of the field radiated by these two contributions plus noise:

(3) $$\begin{array}{c} Y^{m}=A\left(X^{AUT}+X^{s}\right)+Y^{n} \end{array}$$

wherein A is the radiation operator, i.e., the operator mapping the equivalent current matrix on Σ into the matrix collecting the field on Ω [21] and $Y^{n}$ is the matrix collecting the measurement noise contribution at the receiver.

The differential measured matrix is consequently:

(4) $$\begin{array}{c} \hat{Y}=Y^{m}-Y^{GOLD}=A\left(X+X^{s}\right)+Y^{n} \end{array}$$

Rigorously, in order to distinguish the sparse contribution of the AUT and the low rank contribution of the interference source on Σ, the following problem must be solved:

(5) $$\begin{array}{c} \min\;\;\mathrm{rank}\left(X^{s}\right)+\alpha {∥X∥}_{0}\\ \mathrm{subject}\;\mathrm{to}{∥A\left(X+X^{s}\right)-\hat{Y}∥}_{2}\leq \epsilon \end{array}$$

wherein rank(X) is the rank of the matrix *X*, $∥X^{s}{∥}_{0}$ is the $\ell_{0}$ norm of the matrix $X^{s}$, α is a regularization parameter and ϵ depends on the level of the noise affecting the data.

Since both rank minimization and $\ell_{0}$ minimization are non convex functions the solution of (5) requires a computational expensive exhaustive search. Furthermore, $\ell_{0}$ norm is instable in presence of noise.

In order to solve the problem it is advantageous to substitute the original problem with a suitably relaxed version.

In particular, the $\ell_{0}$ norm can be substituted by the $\ell_{1}$ entrywise matrix norm [24],

(6) $$\begin{array}{c} {∥X∥}_{1}=\sum_{k,h}\left|x_{k,h}\right| \end{array}$$

wherein $x_{k,h}$ is the (k,h) entry of the matrix X, while the rank function can be well approximated by the trace norm (also called Schatten 1-norm or nuclear norm) [25,26]:

(7) $$\begin{array}{c} {∥X^{s}∥}_{*}=\sum_{k=1}^{r}\sigma_{k} \end{array}$$

where $\sigma_{k}$ is the *k*-th singular value of $X^{s}$ and *r* is its rank [25,27].

**Figure 4:**
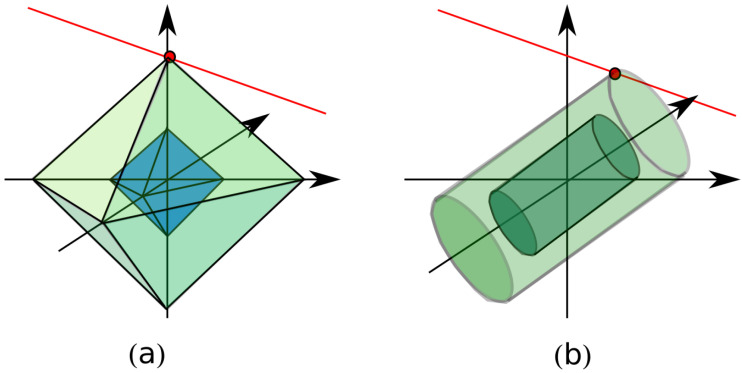
(**a**) geometrical picture of the $\ell_{1}$ minimization; (**b**) geometrical picture of the trace norm minimization.

It is interesting to note that nuclear norm and $\ell_{1}$ norm have some similarities since in some way the nuclear norm is to the rank functional what the convex $\ell_{1}$-norm is to the $\ell_{0}$-norm in the sparse recovery area, Figure 4b. In fact, the nuclear norm can be seen as a relaxed version of the rank norm, while the $\ell_{1}$ norm can be considered a relaxed version of the $\ell_{0}$ norm. While $\ell_{0}$ norm counts the number of elements different from zero, the $\ell_{1}$ norm sums up their amplitude. In the same way, while the rank function counts the number of non-zero singular values, the nuclear norm sums their amplitude. In order to clarify this point, let us recall that in sparse recovery the goal is to identify the sparsest vector (i.e., the vector having the largest number of null components) compatible with the available data [24]. This requires to minimize the so-called $\ell_{0}$ norm, wherein $\ell_{0}$ is the number of non null elements of the unknown vector. Such a minimization is a challenging non convex problem. For the sake of simplicity, let us consider a 3 entries vector, x={x,y,z}. The vector is supposed to be 1-sparse, i.e., only one of the three entries of the vector is different from zero. Let us consider the convex hull of the 1-sparse vectors. Such a convex hull turns out to be the unit ball of the $\ell_{1}$ norm, wherein the $\ell_{1}$ norm is ${∥x∥}_{1}=\left|x\right|+\left|y\right|+\left|z\right|$. A graphical picture of the unit $\ell_{1}$ ball is drawn in Figure 4a. The solution of the $\ell_{1}$ minimization (red point in Figure 4a) is the tangent point between the affine space associated to the available data (drawn as a red line in Figure 4a) and the scaled convex hull. The minimization of the trace norm works in the same way, but operating on the singular values of the matrix.

Consequently, in practice the solution of the problem requires the following minimization procedure involving two different definitions of matrix 1-norm:

(8) $$\begin{array}{c} \min\;\;\alpha ∥X^{s}{∥}_{*}+{∥X∥}_{1}\\ \mathrm{subject}\;\mathrm{to}{∥A\left(X+X^{s}\right)-\hat{Y}∥}_{2}\leq \epsilon \end{array}$$

i.e., a weighted minimization of the Schatten 1-norm (i.e., the trace norm) of $X^{s}$ and of the entrywise 1-norm of X. The regularization parameter α can be estimated using the L-shape curve adopted also in Tikhonov regularization [28].

The above minimization is a convex problem and can be solved by means of the powerful and efficient algorithms available in many numerical libraries.

The algorithm can handle also multiple scattering interference sources. In this case, the field of each interference source gives a low rank matrix on the observation plane. Consequently, the problem is to identify a set of low-rank matrices and a sparse matrix. i.e.,

(9) $$\begin{array}{c} \min\;\;\sum_{l=1}^{L}\alpha_{l}∥X_{l}^{s}{∥}_{*}+{∥X∥}_{1}\\ \mathrm{subject}\;\mathrm{to}{\left|\left|A\left(X+\sum_{l=1}^{L}X_{l}^{s}\right)-\hat{Y}\right|\right|}_{2}\leq \epsilon \end{array}$$

where in *L* is the number of interference sources and $X_{l}^{s}$ is the low rank matrix associated to the *l*-th interference source.

## 4. Numerical Examples

**Figure 5:**
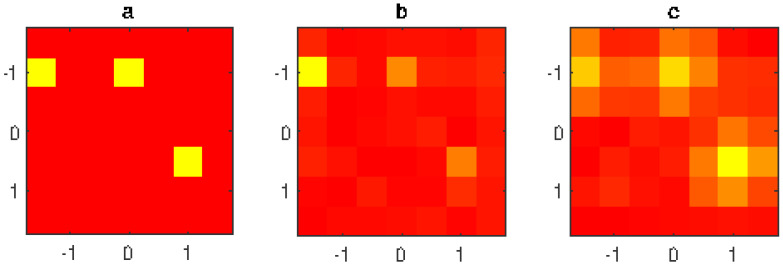
1st example: normalized excitation amplitude of the radiating elements (linear scale in false colors: yellow = null amplitude, red = unit amplitude); (**a**) exact array excitations; (**b**) excitations obtained without filtering; (**c**) excitations obtained using the proposed filtering method; 7×7 planar array with λ/2 inter-element distance, 21×21 measurement points, d=7λ, measured data affected by interference field radiated by a source placed at (x=0,y=2.2λ,z=−8λ) and by −45 dB level Gaussian noise.

In this section some numerical results are shown. The AUT is a 7×7 planar array with λ/2 inter-element distance, centered on the x,y plane of a Cartesian coordinate system (see Figure 3). The data are collected on a 21×21 points λ/2 uniform grid placed on the plane Ω at distance d=7λ from the AUT aperture plane. An undesired source is placed at {x=0,y=2.2λ,z=−8λ}. The data are affected by −45 dB level Gaussian noise. We suppose that the AUT is affected by three fault elements. The excitation of the non fault elements is one, while the three fault elements have zero excitation. The amplitude of the excitations of the 49 radiating elements of the AUT are plotted in Figure 5a (left figure) in false colors (1 = red, 0 = yellow).

The proposed filtering technique is applied to the measured data. The amplitude of the array excitations is shown in Figure 5c (right figure). The three defects are clearly visible.

As a comparison, the same data have been elaborated using the method [5] consisting of $\ell_{1}$ minimization without filtering procedure. The result is plotted in Figure 5b (central figure), showing a less effective identification of the failures.

In particular, the presence of the undesired source makes two broken elements barely identifiable, while the proposed technique is able to clearly identify all three elements.

**Figure 6:**
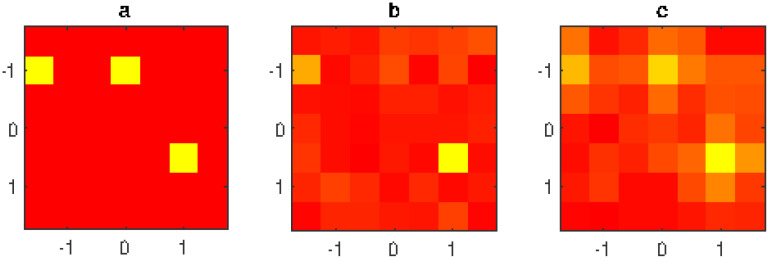
2nd example;: normalized excitation amplitude of the radiating elements (linear scale in false colors: yellow = null amplitude, red = unit amplitude); (**a**) exact array excitations; (**b**) excitations obtained without filtering; (**c**) excitations obtained using the proposed filtering method; 7×7 planar array with λ/2 inter-element distance, 21×21 measurement points, d=7λ, measured data affected by interference field radiated by a source placed at (x=0,y=2.2λ,z=−8λ) and by −35 dB level Gaussian noise.

The estimation algorithm is also stable compared to the noise level. For example, in Figure 6 the estimation of the failures of the AUT is shown in case of −35 dB noise level. The figure shows that the proposed method still gives acceptable results (Figure 6c, right figure), while the standard method fails to identify at least one failure (Figure 6b, central figure).

**Figure 7:**
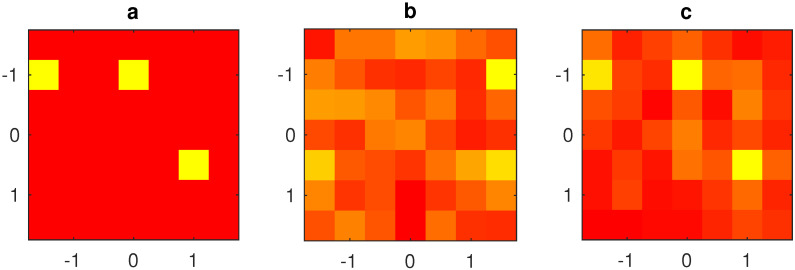
3rd example: normalized excitation amplitude of the radiating elements (linear scale in false colors: yellow = null amplitude, red = unit amplitude); (**a**) exact array excitations; (**b**) excitations obtained without filtering; (**c**) excitations obtained using the proposed filtering method; 7×7 planar array with λ/2 inter-element distance, 21×21 measurement points, d=7λ, measured data affected by interference field radiated by a source placed at (x=0,y=3.2λ,z=−8λ) and a source placed at (x=4,y=0λ,z=−10λ). The data are corrupted by −45 dB level Gaussian noise.

In order to show the performance of the algorithm in case of multiple interference sources, two sources placed at (x=0,y=3.2λ,z=−8λ) and (x=4,y=0λ,z=−10λ) are considered. The data are corrupted by −45 dB level Gaussian noise The solution using the filtering technique is shown in Figure 7c, while the solution not implementing the filtering method is shown in Figure 7b, confirming again an improvement in the estimation of the differential excitation.

**Figure 8:**
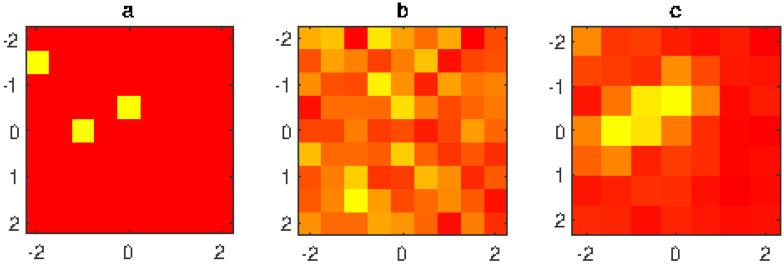
4th example: normalized excitation amplitude of the radiating elements (linear scale in false colors: yellow = null amplitude, red = unit amplitude); (**a**) exact array excitations; (**b**) excitations obtained without filtering; (**c**) excitations obtained using the proposed filtering method; 9×9 planar array with λ/2 inter-element distance, 21×21 measurement points, d=7λ, measured data affected by interference field radiated by a source placed at (x=0,y=3.2λ,z=−8λ) and by −45 dB level Gaussian noise.

Finally, an example of identification of the failures in a larger array (81 radiating elements) is reported in Figure 8. The plot shows that standard technique completely fails to identify the fault elements, while the proposed technique is able to identify the area where two fault elements are placed. The position of the third fault element is not detected, but the figure shows a variation of the excitations on the left upper corner of the array.

In Table 1 the results of the simulations are briefly compared in a quantitative way. As figure of merit, the Mean Square Error between the reference differential amplitude excitations of the AUT and the amplitude excitation of the retrieved differential excitations are reported using the interference source filtering algorithm (4th column and using standard algorithm (5th column of the Table). The CPU time required by the filtering algorithm is reported in the 6th column. Loosely speaking, also the quantitative parameter chosen shows an improvement in the differential excitation reconstruction using the proposed algorithm. The improvement becomes more relevant increasing the number of interference sources. For example, in the case of two sources, the MSE decreases from 1.5 dB to −3.7 dB. Even if this improvement is numerically lower than the 1 source case, it is practically much more relevant, and allows to pass from no failure detection to an effective failure detection, as shown in the previous section. Regarding the computation time, the examples were obtained on an Mc Air 11’ with i7 processor using CVX using only one core. The computation time is less than two minutes, and increases almost linearly with the number of interference sources. However, these values are only indicative, and can give an erroneous idea of the computational time required in real applications.

## 5. Conclusions

In this paper a novel filtering algorithm of signals in planar near-field measurements is described. The method allows the filtering strategy of interference sources in array diagnosis. The technique is simple and numerically efficient, since it allows the use of convex minimization procedures.

The basic idea is to take advantage of the characteristics of the electromagnetic propagation in terms of ‘spreading’ of the field distribution. Briefly, the equivalent current distribution on the array is strongly concentrated on the radiating elements, while the contribution of interference sources placed far from the plane of the array is smoother. Accordingly, it is possible to distinguish these two contributions looking for a ‘sparse’ distribution and a ‘low rank’ distribution. Numerical examples carried out in some simple cases confirm the effectiveness of this approach.

As discussed in the paper, the method proposed in this paper strictly resembles the methods used in image processing. In practice, the interference-affected near field measured on a surface is treated as a (complex-data) image. This allows to use some modern image processing algorithms. In particular, two strategies widely used in image processing are applied in this paper. The first one is the reduction the amount of information by acquiring only the innovation part of an image, as currently happens in video processing, using differential measurements. The second technique has been recently proposed in video denoising and splits the image into a high-sparse and high-rank part.

The results have been obtained using a small laptop computer and CVX program. As stressed in the Introduction, the aim of this paper is to introduce the technique, and for this purpose the computer and program adopted are acceptable. However, CVX is a slow program developed mainly for research-stage applications. More powerful and efficient algorithms are available under payment. The use of these algorithms on powerful parallel computers drops the computational time drastically. Even if computational time is not an issue in near-field measurements, since it is usually a fraction compared to the time required for data acquisition, the possibility of fast reconstruction opens the thrilling possibility of online failure identification, i.e., identification of failures on-site while antenna works [29], filtering the environmental noise of the site where the antenna is placed. This interesting possibility encourages to continue the investigation on the rank properties of the field in the framework of antenna measurements.

References

## Figures and Tables

**Table 1:** First column: number of the example; second column: number of the elements of the AUT; third column: number of interference sources; fourth column: Mean Square Error of the amplitude of the differential excitations using the proposed technique; fifth column: Mean Square Error of the amplitude of the differential excitations without using the proposed technique; sixth column: CPU time (seconds) required by the filtering program.

Example	Array Elements	Number of Interf.	MSE Filt	MSE no Filt.	CPU Time
1st	7 × 7	1	−7.5 dB	−3.7 dB	95 s
2rd	7 × 7	1	−9.5 dB	−3.7 dB	77 s
3rd	7 × 7	2	−3.7 dB	1.5 dB	167 s
4rth	9 × 9	1	−4.8 dB	3.9 dB	78 s
